# The Use of Voice Assistant for Psychological Assessment Elicits Empathy and Engagement While Maintaining Good Psychometric Properties

**DOI:** 10.3390/bs13070550

**Published:** 2023-07-02

**Authors:** Stefania Mancone, Pierluigi Diotaiuti, Giuseppe Valente, Stefano Corrado, Fernando Bellizzi, Guilherme Torres Vilarino, Alexandro Andrade

**Affiliations:** 1 Department of Human Sciences, Society and Health, University of Cassino and Southern Lazio, 03043 Cassino, Italy; s.mancone@unicas.it (S.M.); giuseppe.valente@unicas.it (G.V.); stefano.corrado@unicas.it (S.C.); fernando.bellizzi@unicas.it (F.B.); 2 Health and Sports Science Center, Department of Physical Education, Santa Catarina State University, Florianópolis 88035-901, Brazil; guilherme.vilarino@udesc.br (G.T.V.); alexandro.andrade.phd@gmail.com (A.A.)

**Keywords:** vocal assistant, conversational agents, psychometric assessment, empathy, interpersonal reactivity, engagement, measurement invariance, artificial intelligence

## Abstract

This study aimed to use the Alexa vocal assistant as an administerer of psychometric tests, assessing the efficiency and validity of this measurement. A total of 300 participants were administered the Interpersonal Reactivity Index (IRI). After a week, the administration was repeated, but the participants were randomly divided into groups of 100 participants each. In the first, the test was administered by means of a paper version; in the second, the questionnaire was read to the participants in person, and the operator contemporaneously recorded the answers declared by the participants; in the third group, the questionnaire was directly administered by the Alexa voice device, after specific reprogramming. The third group was also administered, as a post-session survey, the Engagement and Perceptions of the Bot Scale (EPVS), a short version of the Communication Styles Inventory (CSI), the Marlowe–Crowne Social Desirability Scale (MCSDS), and an additional six items to measure degrees of concentration, ease, and perceived pressure at the beginning and at the end of the administration. The results confirmed that the IRI did keep measurement invariance within the three conditions. The administration through vocal assistant showed an empathic activation effect significantly superior to the conditions of pencil–paper and operator-in-presence. The results indicated an engagement and positive evaluation of the interactive experience, with reported perceptions of closeness, warmth, competence, and human-likeness associated with higher values of empathetic activation and lower values of personal discomfort.

## 1. Introduction

Psychotherapy and clinical psychology are beginning to experiment with the possibilities of using and integrating new technologies in a clinical setting. Cognitive behavioral therapy has been the most experimented-upon, and several studies have shown that the traditional in-person method and computer-based intervention are equivalent as to their results, while some studies have also shown the greater effectiveness of distance intervention [1,2,3,4].

Artificial intelligence, deep learning and machine learning have increased the technological level of chat bots, which have evolved from text programs into multimedia and conversational programs, providing a multimedia and multisensory augmented-reality experience. Applications in the medical and healthcare sector in general, including clinical psychology and mental health, have gradually become capable of involving the user in a virtual relationship, increasing the level of participation and compliance to therapeutic treatment, and improving psychological interventions in general [5,6,7,8].

The idea of a human interaction or conversation with a machine makes it easier and more stimulating to search for information in people, even when there is awareness of the fact that the interaction takes place with a computer program that simulates a human being and cannot independently generate information that has not been programmed and preset. In confirmation of the cognitive paradox that is created between reacting to the provocations of the robot machine as if there were a real interaction with a person, and the awareness that the machine is programmed in this way and has no intentionality, there are two studies that have highlighted the effectiveness of “trash talk” and “negative feedback” in altering strategy, perception, and provoking an emotional reaction, even when a robot is “talking” [9,10].

Such technological progress and the increasing diffusion of robotic tools and software also begin to stimulate ethical reflection on the use of technology in the field of clinical psychology and mental health [11]. The importance of the therapist in the intervention with the patient is well known; however, the creation of new technologies that allow for assistance and collection of information about the patient’s situation is fundamental in order to facilitate the treatment. With the current situation relating to the pandemic caused by COVID-19, strategies for remote assistance and information collection are essential for monitoring the population and for creating coping policies [12,13].

The diffusion of interactive technological devices with a voice-assistant function (satellite navigators first, and then portable multimedia devices and smartphones with voice search function and interactive toys, as well as devices such as Alexa, Siri, Cortana and Google Now), which use voice as a stimulus to attract attention and engage the user, has been extensive. The voice is a powerful natural stimulus able to capture attention and engage us emotionally, even within the technological tools used in the field of mental health [14].

Although applications, chat bots, voice assistants, and virtual assistants are present in the field of health, we are still in an experimental phase focused on the collection of data for building the theoretical basis of technological intervention in mental health. There is certainly a need to define the effectiveness of these instruments through a search for solid empirical evidence and the promotion of more independent studies. So far, a certain limit has been represented by the fact that most publications have been written by the authors of computer programs themselves [15]. At the moment, however, the results seem promising and favorable [16,17]. Health psychology could draw useful information from the extensive research on human–computer interaction to improve the tools available.

Anthropomorphism studies provide useful explanations and information on the ability of many humans to attribute human characteristics to inanimate objects [18]. By referring to a human-like voice as opposed to a synthetic or robotic voice, some researchers have started to look into how people perceive voice assistants as being anthropomorphic [19]. Other scholars have analyzed the humanity of voice assistants, which is the general perception of the voice assistant as a human interlocutor. The humanity of voice assistants has been evaluated using various measurement scales (for example, social presence, human-likeness, and perceived humanness) [20,21,22,23]. Several assistive solutions using conversational agents are spreading in the health sector (e.g., AIVA, ORBITA AI, and OMRON) [24,25,26].

Bèrubè et al. [27] recently proposed a systematic review of studies on voice-based conversational agents for the prevention and management of chronic and mental conditions. The included studies were between 2009 and 2020, but most of the papers were published in 2019. The survey included 12 papers reporting studies on the development and evaluation of conversational speech agents in terms of system accuracy and technology acceptance [28,29,30,31,32,33,34,35,36,37,38,39]. System accuracy refers to how well the voice conversational agents can interact with the users, either in terms of how well they can recognize speech or how well they can answer users’ questions. Acceptance of technology refers to all aspects of the user’s perception of the system (for instance, user experience, ease of use, and efficiency of interaction). Three papers in total have addressed psychological conditions. They concentrated specifically on depression, intellectual disability, dementia, and autism. The interventions were classified as monitoring, support, or both. Monitoring interventions are those that focus on health tracking (such as symptoms and medication adherence), whereas support interventions include information or alerts that are targeted or on-demand. All of these studies were non-experimental, and the evaluation methods varied widely, particularly in terms of users’ perception of the technology (for example, user experience).

Existing digital therapy formats typically emphasize psychoeducation and a modular style, one with fixed content and duration, which is inflexible for users [40,41]. In comparison to other digital mental health interventions, conversational agents offer greater interactivity, which simulates therapeutic conversation, as well as choice and control over session content and intensity. Research has shown that users respond and interact with conversational agents in social ways, and that the agents can encourage openness [42,43]. Focusing upon the conditions under which humans feel willing to share information, a recent study, howewer, highlighted the issue of the willingness of respondents to participate in alternative modes of data collection. There appeared to be significant differences in willingness to participate, according to demographic characteristics. Age was found to be the most decisive basic characteristic for participation. The older the respondents, the more likely they were to participate in personal interviews [44].

Conversational agents have diverse applications in mental health, including diagnostic tools, symptom monitoring, and treatment or intervention [45]. The systematic review by Gaffney et al. [46] illustrates thirteen promising papers published from 2013 to 2018 on the use of conversational agents for treatment of mental health problems [47,48,49,50,51,52,53,54,55,56,57,58]. The conversational agents included natural language input, either written or spoken, and fixed on-screen response options for participant responses. They provided interventions designed to reduce symptoms, improve well-being, and enhance self-management. Repetitive content, limitations in the agent’s ability to comprehend or respond appropriately, a shallow or superficial relationship, and the sound and quality of the agent’s voice were the most frequently cited obstacles to successful intervention with a conversational agent [46]. However, overall, there is interesting evidence on the present effectiveness and possible future developments in the uses of such tools for the purposes of psychological intervention.

Another recent specific area is the use of the conversational agent for psychological assessment purposes. A diagnostic conversation with a medical professional and a diagnostic conversation with a conversational agent were compared by Philip et al. [59]. They discovered that, while the conversational agent had high accuracy in diagnosing major depression, the agent had lower accuracy with mild depressive symptoms. Maharjan et al. presented their conversational agent called Sofia, which was able to administer the WHO-5 well-being scale through spoken conversation [60]. Caballer et al. [61] have recently reported a study in which they present the psychometric comparison of paper–pencil and via-chatbot administration of a scale on loneliness in a sample of college students, highlighting the adequacy of validity and internal consistency of both modalities.

To the best of our knowledge, there are no other studies that have provided psychometric evidence on the validity of administering psychological assessment instruments through a conversational agent. In this regard, the question of the influence of voice on users’ level of attention and involvement in the interaction also arises. This has been reflected in various recent works, papers mostly focusing on commercial, information, or service delivery interactions [62,63,64,65]. Recent qualitative investigations on vocal assistant use suggest that users particularly value the efficiency of speech interaction [66,67]. Evidence reported in Rzepka et al. [68] indicates that speech exhibits higher perceived efficiency, lower cognitive effort, higher enjoyment, and higher service satisfaction than does text-based interaction. Kim et al. [69] discovered that the anthropomorphism of digital assistants influences the warmth and pleasure perceived by users. Warmth captures characteristics associated with the perceived intentions of the social object, such as, but not limited to, trustworthiness, sincerity, and friendliness [70]. The study by Carolus et al. [71] focused on analyzing the empathic impact of smart speakers, describing the technology’s potential to trigger emotional reactions in users. The work of Kim et al. [72] investigated the effect of nonverbal vocal cues in speech interactions on the user’s perception toward the agent. While working within the perspective of device design, Yalçın presented a framework to equip conversational agents with real time multi-modal empathic interaction capabilities, including basic dialogue capabilities, as well as three levels of empathic behavior in a conversational scenario [73].

In light of this framework of recent literature, and considering the need to produce further evidence on the psychometric validity of assessments conducted using conversational agents, the main objective of our present study was to use the Alexa vocal assistant, specifically an Amazon Echo device, as a real psychologist’s assistant able to administer psychometric tests to a sample of subjects, while assessing the efficiency and validity of this measurement. To this end, a specific skill has been developed as an application of Alexa’s software capable of vocally administering psychological rating scales. Careful preliminary programming of Alexa’s dialogic interaction was carried out during the skill construction phase. Since the aim was to avoid the result of simple and rigid vocal repetition of the succession of test questions, special attention was paid to the inclusion of a colloquial tone for the dialogic passages and a function preparatory to the presentation of the scales. Some expressions were identified and inserted for the specific purpose of demonstrating an attention to the interlocutor, for example where it was said that, in case of doubt or misunderstanding, he could at any time ask for a repetition of the sentence. Particularly smooth and, so to speak, “accompanied” were the transitions from one scale to another.

Given the level of emotional involvement induced by the use of the conversational agent, it was considered important to produce a comparison of the interpersonal and empathic activation produced by the traditional paper–pencil test administration methodology, that produced by a mode of reading and collecting self-report assessments through an in-person administrator, and that produced by administration through reprogramming of the Alexa conversational agent. For this purpose and for verification of measurement invariance, the brief version of the Interpersonal Reactivity Index (IRI) was administered to all participants in paper–pencil mode as a baseline (Pre) and after a week to each group (Post) according to the respective mode: paper–pencil, by operator dictation, and through the use of Alexa. The procedure also included administering to the third group a post-session survey including the Engagement and Perceptions of the Bot Scale (EPVS), the Marlowe–Crowne Social Desirability Scale (MCSDS), a short version of the Communication Styles Inventory (CSI), and in addition, six items to measure degrees of concentration, ease, and perceived pressure at the beginning and at the end of the administration. There were therefore five main hypotheses that guided our work (some of them articulated in further sub-hypotheses): (1) The administration of psychometric measurement through Alexa would lead to equally reliable assessment with respect to the self-report paper–pencil and the assistance with reading by an operator in their presence. (2) The interaction with the conversational agent would solicit higher empathic activation than would the other two modes of delivery. (3) The interaction with the vocal device would: (a) not compromise their ability to concentrate; (b) made them feel more comfortable; and (c) progressively ease the feeling of pressure exerted by the unusual situation. (4) The higher the interpersonal reactivity, the higher the (a) perceived engagement; and (b) level of positive evaluation of the interactive experience with the conversational agent Alexa would be. (5) Engagement and positive evaluation of the interactive experience with the vocal assistant would also show significant associations with (a) a disposition for social desirability, and (b) a communication style characterized by a prevalence of emotional connotations.

## 2. Materials and Methods

### 2.1. Development of the Software Application

**Figure 1:**
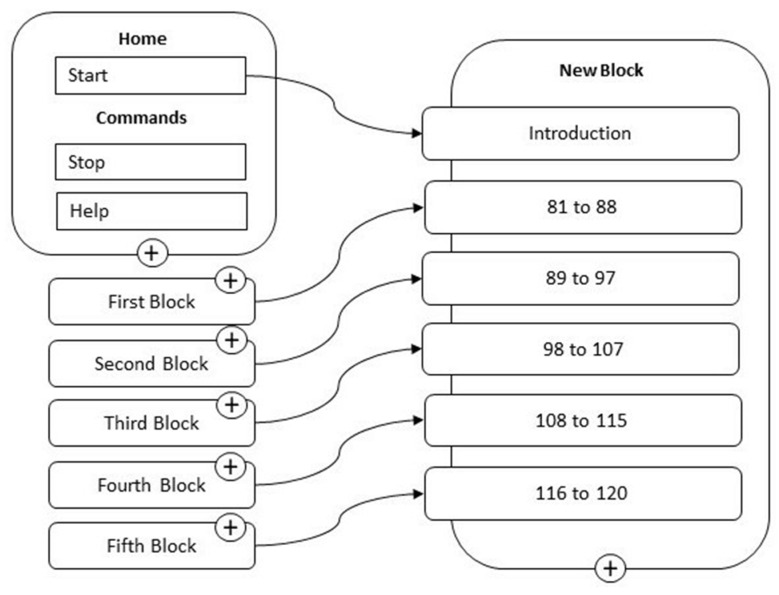
Block diagram.

In order to achieve the purpose of the study, a skill, a software application from Alexa, was designed and created on which the different scales of the test were loaded. The skill was developed on the portal dedicated to Amazon Developer programmers and through the Alexa Developer Console. In addition, to facilitate programming and make the skill more efficient, the voice assistant programming portal Voiceflow.com was used to design and build the voice interface visually, without the need to know the specific programming codes such as, for example, in this particular case, json, an acronym for javascript object notation, a format suitable for exchanging data between client/server applications. The usefulness of Voiceflow, in addition to simplified software development, lies in the community created by the Canadian company, with tutorials, discussion groups of programmers from all parts of the world, and fast and efficient online assistance, which collectively make it possible to program rather quickly and effectively solve the problems that normally arise during complex software development. The novelty introduced here has been to overturn the roles and transform the common idea of the vocal assistant to which the user asks questions in order to have answers and solutions, into the one (given the female voice) who asks questions to which the user must answer. Below in Figure 1 is the overall block diagram that was developed.

**Figure 2:**
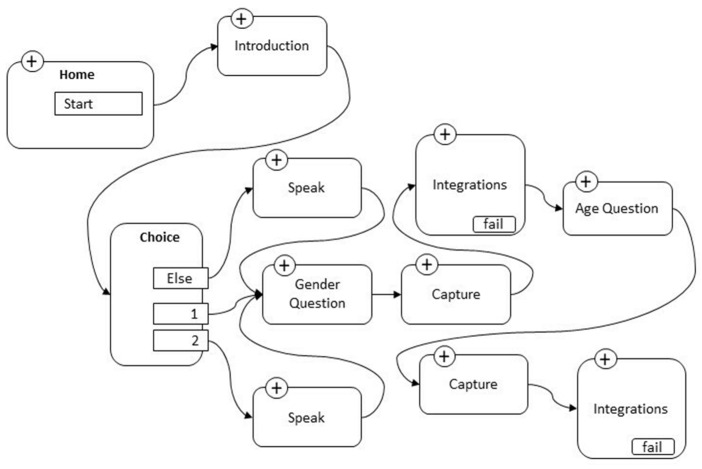
Sequence from the capture block to the integration block.

The skill has been developed to be started by the user with the phrase “Alexa, start Empathy test”. After the introduction, in which Alexa explains how the test works before proceeding to the age and sex request, the actual administration starts. The users’ voice responses, chosen from a list of options dependent on the relative blocks (judgments such as ‘little’, ‘enough’, and ‘completely’; choices between true or false; and grades from 1 to 5), were acquired by the device (capture block) and sent in real time, through the integrations block, to a spreadsheet. The user’s voice responses were then captured, transformed into text, and automatically transcribed into the relevant rows and columns in the dedicated spreadsheet created in GoogleSheet. At the end of the administration work, an Excel file with all the answers automatically acquired by the system was obtained. The ratings were transformed into numbers in order to create a matrix that could be used with statistical analysis software. Figure 2, below, shows the process sequence from the capture block to the integration block.

### 2.2. Participants Selection and Administration Procedure

The overall sample used for the study consisted of 300 university students, aged between 18 and 31, who freely took part in the study after being openly invited during class sessions of the Personality and Sport Psychology courses. Voluntary participation in the study was formally initiated through a specific link distributed to students through which they indicated their intention to participate by entering their matriculation number and email address to be contacted later. In the presentation of the study, it was specified that no recognition in either money or university credits would be provided. Tools administration took place upon release and signing of the form for an informed consent of participation. Collections occurred in the laboratory of Epidemiology, Motor Activity and Lifestyles of the University. The local IRB approved the study protocol. The participants were randomly divided into three groups of 100 participants each. After an initial administration to all participants, with the usual paper–pencil mode, of the scale chosen for psychometric and empathic activation testing (baseline measure), the same instrument was administered after one week, but in the first group by means of a paper version, in the second group read by a person in attendance, with the operator recording the answers declared by the participants at the same time, and in the third group by means of the Alexa voice device. Each administration lasted on average 20 min. The procedure also included administering to the third group a post-session survey (by paper–pencil mode) including the Engagement and Perceptions of the Bot Scale (EPBS), the short version of the Communication Styles Inventory (CSI-B), the Marlowe–Crowne Social Desirability Scale (MCSDS) and, in addition, an index to measure degrees of concentration, ease, and perceived pressure at the beginning and at the end of the administration.

### 2.3. Tools

The scales selected for the administration were the following:

(1) Brief Version of Interpersonal Reactivity Index (IRI-B) [74,75,76]. The instrument includes a self-reported scale of 16 items with a 5-point Likert scale response, from “Doesn’t describe me well” to “Describes me very well”. The four sub-factors are: (A) perspective taking, the tendency to spontaneously adopt the psychological point of view of others; (B) imagination, drawing on respondents’ tendencies to transpose themselves imaginatively into the feelings and actions of fictitious characters in books, films and plays; (C) concerns/empathetic activation, which assesses feelings of sympathy “other-oriented” and concern for unfortunate other people; and (D) personal discomfort, which measures “self-oriented” feelings of personal anxiety and discomfort in tense interpersonal contexts. Reliability measures by paper–pencil mode for this study were, respectively, for perspective taking: α = 0.71 [CIs 95% 0.685; 0.765]; ω = 0.72 [CIs 95% 0.683; 0.771], for imagination: α = 0.74 [CIs 95% 0.696; 0.795]; ω = 0.75 [CIs 95% 0.701; 0.799], for concerns/empathetic activation: α = 0.71 [CIs 95% 0.666; 0.767]; ω = 0.72 [CIs 95% 0.661; 0.770], and for personal discomfort: α = 0.72 [CIs 95% 0.668; 0.771]; ω = 0.73 [CIs 95% 0.678; 0.778].
(2) Marlowe–Crowne Social Desirability Scale (MCSDS) [77]. The Italian short version with 9 true/false response items was used [78]. The instrument is widely used to assess and control for response bias in research with self-report requests. The scale was developed to measure social desirability, defined as an individual’s need to gain approval by responding in a culturally appropriate and acceptable manner. Participants were requested to respond to each item on a 7-point scale ranging from 1 = Absolutely false to 7 = Absolutely true. A total score is derived from the sum of all items, ranging from 7 to 91. Higher scores indicate higher levels of social desirability. Reliability measures by paper–pencil mode for this study were: α = 0.76 [CIs 95% 0.735; 0.795]; ω = 0.77 [CIs 95% 0.743; 0.798].
(3) The short version of the Communication Styles Inventory (CSI-B) [79] contains 18 items, which in turn converge separately on three factors. The first factor measures the ability of the person to exercise effective impression manipulativeness during the conversation. The person who has charm attracts attention and involves people, despite their own will. This mode of communication is based on the pleasure of others, so that they are well prepared to accept the requests that are addressed to them. The second factor, described as emotionality, refers to the emotional activation produced in the individual as a result of verbal interaction. An emotional transport tends to accompany the person’s conversation, which with difficulty contains their emotions, both when the subject is a current matter and when it relates to stories that refer to the person’s past. The individual has a pronounced empathic capacity, so that the intense emotional states of others do not leave him/her indifferent, rather he/she tends to try to identify with the emotions of others. The third factor, referred to as expressiveness, refers to the individual’s ability to be effective in conversation by monitoring and balancing the elements of communication, such as the quality of the topic illustrated, as supported with an adequate amount and variety of data and sources; the clear and consistent presentation of the topic to keep interest and attention alive; the enhancement of non-verbal resources, such as posture, gestures, eye contact, pauses and silences; the ability to reposition the speech if it deviates; and not least, a shrewd management of the available time. Reliability measures by paper–pencil mode for this study were, respectively, for impression manipulativeness: α = 0.75 [CIs 95% 0.714; 0.790]; ω = 0.74 [CIs 95% 0.705; 0.801], for emotionality: α = 0.81 [CIs 95% 0.757; 0.855]; ω = 0.80 [CIs 95% 0.751; 0.869], and for expressiveness: α = 0.76 [CIs 95% 0.712; 0.793]; ω = 0.75 [CIs 95% 0.720; 0.782].
(4) Engagement and Perceptions of the Bot Scale (EPBS), an adaptation from Liang et al. [80] containing 18 items, consists of users’ self-reported ratings with 5-point Likert scales on two dimensions: user engagement and the participant’s perception of the bot (which includes five constructs: perceived closeness, perceived bot warmth, perceived bot competence, perceived bot human-likeness, and perceived bot eeriness). Engagement hints at how much people enjoy the conversation, which is an essential indication of people’s willingness to continue the conversation. It was measured through three items: (1) How engaged did you feel during the conversation? (2) How enjoyed did you feel during the conversation? (3) How interested did you feel during the conversation? Reliability measures by paper–pencil mode were: α = 0.81 [CIs 95% 0.765; 0.841]; ω = 0.80 [CIs 95% 0.753; 0.837]. Closeness was measured using three items as well, and considers that a close relationship is often built by self-disclosure behavior: (4) How close did you feel with the bot? (5) How connected did you feel with the bot? (6) How associated did you feel with the bot? Reliability measures by paper–pencil mode were: α = 0.75 [CIs 95% 0.715; 0.780]; ω = 0.75 [CIs 95% 0.703; 0.776]. Warmth was measured through three items, which inquired as to how friendly/sympathetic/kind the participants deemed the bot: (7) How friendly did you find the bot? (8) How sympathetic did you find the bot? (9) How kind did you find the bot? Reliability measures by paper–pencil mode were: α = 0.72 [CIs 95% 0.691; 0.740]; ω = 0.71 [CIs 95% 0.688; 0.736]. Competence has been included to measure, through three items as well, how participants assessed the bot’s ability to conduct a conversation: (10) How coherent did you feel the conversation? (11) How rational did you feel the conversation? (12) How reasonable did you feel the conversation? Reliability measures by paper–pencil mode were: α = 0.70 [CIs 95% 0.674; 0.737]; ω = 0.70 [CIs 95% 0.682; 0.733]. Human-likeness was included to understand to what degree participants perceived the bots as humans: (13) How human-like did you find the bot? (14) How natural did you find the bot? (15) How lifelike did you find the bot? Reliability measures by paper–pencil mode were: α = 0.73 [CIs 95% 0.675; 0.754]; ω = 0.72 [CIs 95% 0.673; 0.742]. Eeriness has been included to see whether participants thought that the bot was weird: (16) How weird did you find the bot? (17) How creepy did you find the bot? (18) How freaked out were you by the bot? Reliability measures by paper–pencil mode were: α = 0.70 [CIs 95% 0.681; 0.741]; ω = 0.70 [CIs 95% 0.679; 0.754].
(5) Index of Concentration, Ease and Perceived Pressure (ICEPP). At the beginning and end of the Alexa administration, participants were asked a further 6 short questions (with answers on a 5-point scale, from 1 (not at all) to 5 (very much)), related to the degrees of concentration, ease, and perceived pressure at the beginning and at the end of the administration: (1) How concentrated do you feel at the beginning of this test? (2) How concentrated did you feel at the end of the test? (3) How much pressure do you feel at the beginning of this test? (4) How much pressure did you feel at the end of the test? (5) How comfortable do you feel at the beginning of the test? (6) How comfortable did you feel at the end of the test?

### 2.4. Statistical Analysis

The data were processed using IBM SPSS software version 26. Crombach’s alpha and McDonald’s omega were considered as measures of scale reliability. In order to compare differences in the interpersonal reactivity as a result of the three different modes of administration compared with a common initial baseline administration by paper-matrix, a two-way repeated measures ANOVA was run with two independent variables (method of administration, time) and one dependent variable (each subscale measuring interpersonal reactivity). The primary purpose was to understand if there is an interaction between these two factors and the dependent variable. As the number of participants in the groups was balanced, in order to determine the interaction between the variables, Pillai’s criterion was used instead of Wilks’ Lambda, as it is more robust to unequal covariance matrices [81]. Normality of data was assessed by the Shapiro–Wilk test. The homogeneity of the three samples with respect to the genre was assessed with the chi-square test with a significance level of *α* < 0.05. Following Cohen [82], partial Eta squared (ηp^2^) was the measure used to assess effect size (0.01 = small, 0.06 = medium, 0.13 = large). The level of significance was set at *α* < 0.05, while for the testing of multiple univariate interaction effects, a Bonferroni adjustment was introduced by dividing the declared level of statistical significance by the number of dependent variables: *α* < 0.025 (i.e., *α* < 0.05 ÷ 2). The follow-up investigation proceeded with the computation of simple effects tests in order to reveal the degree to which one factor was differentially effective at each level of the second factor. Pre–post comparisons were made by T-testing matched samples with *α* < 0.05. The assessment of internal consistency of the scales was evaluated through Cronbach’s and McDonald’s coefficients. Pearson’s coefficient was used to explore correlations between the psychometric tools. Measurement invariance of the factorial structure of the IRI by condition of administration was assessed. Three nested models with increasing degrees of restriction were tested: the base model assessed configural invariance and allowed free estimation of all the parameters for each group. The metric (weak) invariance model, nested in the configural model, added the restriction of invariant factor loadings among groups. Finally, the scalar (strong) invariance model, nested in the second model, added the intercept constraint of the invariant items among the comparison groups. Given that the chi-square indices are sensitive to the sample size, we focused mainly on the comparison of the CFI, TLI, and RMSEA indices. To test the adequacy of the CFA model, as suggested by technical literature [83], chi-square, CFI (comparative fit index), TLI (Tucker–Lewis index) and RMSEA (root–mean–square error of approximation) were used as relevant fit indicators, with CFI and TLI > 0.90 and RMSEA < 0.08 as acceptable model-fit indicators [84].

## 3. Results

The participants (N = 300), of whom 134 were male (44.66 %) and 166 female (55.33%), had an average age of 23 years (SD = 4.11). The homogeneity of the three samples considering the gender variable was tested by a preliminary χ^2^ analysis (3 groups × 2 genders), which found no significance. Therefore, the different gender ratios of the groups did not affect their statistical homogeneity.

The measurement invariance of the factorial structure of the brief version of Interpersonal Reactivity Index by condition of administration was then assessed. Three nested models with increasing degrees of restriction were tested. Table 1 shows the goodness-of-fit indices of the model by condition of administration and nested models of invariance in ascending order of restriction level. The results confirmed that the tool showed invariance by the three conditions, and also that the fit of the scale for the third condition of administration was good.

Prior to the subsequent ANOVA two-way analysis, the verification of the Studentized residuals showed that, for all four subscales of the Interpersonal Reactivity Index, there was normality, as assessed by the Shapiro–Wilk test of normality, there were no outliers, as there were no Studentized residuals greater than ± 3 standard deviations, and there was sphericity for the interaction term, as assessed by Mauchly’s test of sphericity (*p* > 0.05).

For the Perspective Taking subscale, there was not a statistically significant interaction between mode and time on Perspective Taking: F(2, 198) = 2.93, *p* > 0.001, partial η^2^ = 0.023. No significance of the effect resulted with regard to the mode variable (*p* > 0.025; ηp^2^ = 0.031) or the time variable (*p* > 0.025; ηp^2^ = 0.002).

For the Imagination subscale, there was not a statistically significant interaction between mode and time on Imagination: F(2, 198) = 0.07, *p* > 0.001, partial η^2^ = 0.007. No significance of the effect resulted with regard to the mode variable (*p* > 0.025; ηp^2^ = 0.011) or the time variable (*p* > 0.025; ηp^2^ = 0.027).

**Figure 3:**
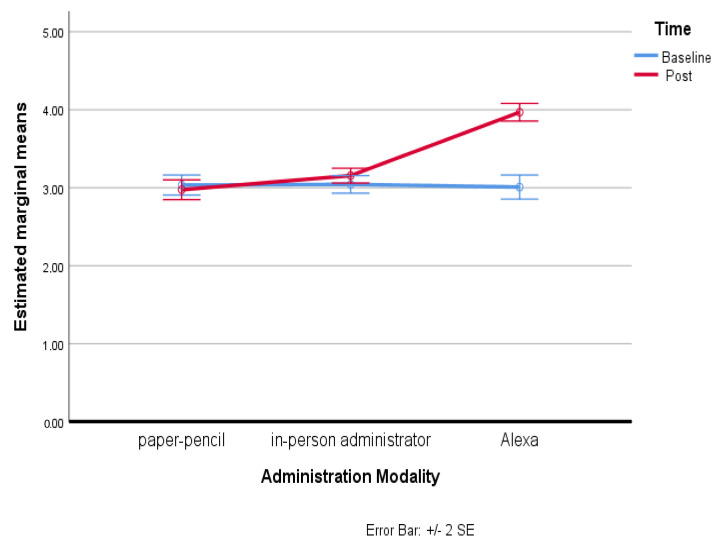
Estimated marginal means of Empathetic Activation.

For the Empathetic Activation subscale, there was a statistically significant interaction between mode and time on Empathetic Activation: F(2, 198) = 39.02, *p* < 0.001, partial η^2^ = 0.283. Significance of the effect resulted with regard to the mode variable (*p* < 0.025; ηp^2^ = 0.282) and the time variable (*p* < 0.025; ηp^2^ = 0.315). Therefore, simple main effects were run: as the participants went through the baseline assessment session, the Empathetic Activation was not statistically significantly different (*p* > 0.05). Specifically, comparing the third group (M = 3.00, SD = 0.77) with the first group (M = 3.01, SD = 0.64) a mean difference of 0.03, 95% CI [−0.25, 0.20] resulted, and comparing the third group with the second group (M = 3.04, SD = 0.57) a mean difference of −0.03, 95% CI [−0.26, 0.19] resulted, F(1, 99) = 0.081, *p* = 0.923, partial η^2^ = 0.001. But as the participants went through the subsequent assessment differentiated by mode of administration, the Empathetic Activation results were statistically significantly different in the group which had received the administration of the instrument through Alexa (third group) (M = 3.97, SD = 0.57) compared to the first group, which had received the administration of the instrument in paper–pencil mode (M = 2.97, SD = 0.63), showing a mean difference of 0.99, 95% CI [0.79, 1.20], as well as to the second group, which had received the administration of the instrument with the in-person administrator (M = 3.15, SD = 0.48), showing a mean difference of 0.81, 95% CI [0.64, 0.90], F(1, 99) = 138.399, *p* < 0.025, partial η^2^ = 0.583. The following graph, Figure 3, shows overall scores in the Empathetic Activation considering both administration mode and the time factor. As can be noted, a significant effect was found in the third group, which had the survey administered by the Alexa voice assistant.

**Figure 4:**
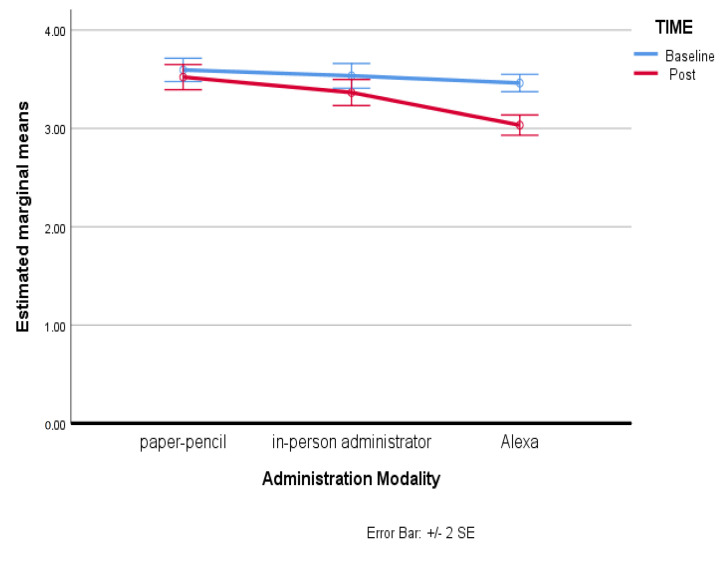
Estimated marginal means of Personal Discomfort.

For the Personal Discomfort subscale, there was a statistically significant interaction between mode and time on Personal Discomfort, F(2, 198) = 15.09, *p* < 0.001, partial η^2^ = 0.089. Significance of the effect resulted with regard to the mode variable (*p* < 0.025; ηp^2^ = 0.120) and the time variable (*p* < 0.025; ηp^2^ = 0.205). Therefore, simple main effects were run: as the participants went through the baseline assessment session, the Personal Discomfort was not statistically significantly different (*p* > 0.05). Specifically, comparing the third group (M = 3.46, SD = 0.44) with the first group (M = 3.59, SD = 0.59) a mean difference of −0.13, 95% CI [−0.31, 0.04] resulted, and comparing the third group with the second group (M = 3.53, SD = 0.63) a mean difference of −0.07, 95% CI [−0.26, 0.11] resulted, F(1, 99) = 3.321, *p* = 0.071, partial η^2^ = 0.032. But as the participants went through the subsequent assessment differentiated by mode of administration, the Personal Discomfort result was statistically significantly different in the group which had received the administration of the instrument through Alexa (third group) (M = 3.03, SD = 0.51), as compared to the first group, which had received the administration of the instrument in paper–pencil mode (M = 3.52, SD = 0.64), with a mean difference of −0.49, 95% CI [−0.70, −0.28], and as compared to the second group, which had received the administration of the instrument with the in-person administrator (M = 3.36, SD = 0.66), with a mean difference of −0.33, 95% CI [−0.54, −0.13], F(1, 99) = 32.758, *p* < 0.025, partial η^2^ = 0.249. The following graph, Figure 4, shows overall scores in the Personal Discomfort considering both administration mode and the time factor. As can be noted, a significant effect was found in the third group, which had the survey administered by the Alexa voice assistant.

Table 2, following, shows the bivariate correlations between IRI’s four subscales (Perspective Taking; Imagination; Empathetic Activation; Personal Discomfort), Marlowe–Crowne Social Desirability Scale (MCSDS), the three subscales of the Communication Styles Inventory (CSI-B/I; Manipulativeness; Expressiveness; Emotionality), and the six subscales that make up the Engagement and Perceptions of the Bot Scale (EPBS; Engagement; Closeness; Warmth; Competence; Human-likeness; Eeriness) evaluated in the group that received the administrations through the Alexa device.

From the table, it can be seen that participants’ perceived level of engagement in interacting with the Alexa device reported positive associations with Empathetic Activation (0.257 **) and with the communication style distinguished by Emotionality (0.324 **), while the association was inverse with Personal Discomfort values (−0.259 **). The perception of Closeness also reported similar trends: positive associations with Empathetic Activation (0.268 **) and with the communication style distinguished by Emotionality (0.301 **), negative associations with Personal Discomfort (−0.310 **). Perception of Warmth showed significant correlation with Empathetic Activation (0.336 **) and again with a communication style distinguished by Emotionality (0.297 **). Perception of Human-likeness was also found to be correlated with Empathetic Activation (0.329 **), with the communication style Emotionality (0.264 **), and also with the communication style characterized by Expressiveness (0.332 **). The perception of Eeriness in interacting with the Alexa device was found to correlate positively with Personal Discomfort (0.277 **) and inversely to the values of Empathetic Activation (−0.268 **). The Social Desirability scale reported positive correlations with Perspective Taking (0.260 **) and with the communication style marked by Expressiveness (0.332 **). Overall, the analysis of the different correlations between variables showed a clear association of Empathetic Activation with a positive and engaging perception of interaction with the device reprogrammed as a psychological test administerer.

Paired-samples t-tests were used to determine whether there was a statistically significant mean difference between participants’ judgments regarding concentration, pressure and ease, as perceived at the beginning and at the end of the device’s administration. The assumption of normality was not violated, as assessed by the Shapiro–Wilk test (*p* = 0.723). The following was found to be the case: (a) The perceived concentration did not vary significantly from beginning to end; M_1_ = 3.97 (SD = 1.01) < M_2_ = 4.19 (SD = 0.87), an increase of 0.220 (95% CI, 0.479 to −0.039), t(99) = −1.683, *p* > 0.05, d = 0.17. (b) Perceived pressure showed a significant decrease in mean scores at the end of the compilation; M_1_ = 2.43 (SD = 1.26) < M_2_ = 1.99 (SD = 1.16), a decrease of 0.440 (95% CI, 0.184 to 0.696), t(99) = 3.412, *p* < 0.001, d = 0.34. (c) Perceived comfort increased significantly (*p* = 0.001) during the compilation; M_1_ = 3.65 (SD = 1.00) < M_2_ = 4.33 (SD = 0.89), an increase of 0.680 (95% CI, 0.911 to 0.449), t(99) = −5.855, *p* < 0.001, d = 0.58. Therefore, for the participants, the interaction with the vocal device did not compromise their ability to concentrate, made them feel more and more comfortable, and progressively eased the feeling of pressure exerted by the unusual situation.

## 4. Discussion

The statistical analysis of the data did confirm the main hypotheses on which the experimental work was built; the results obtained through Alexa have passed the invariance test, and therefore, the vocal assistant can be used for psychometric tests with the certainty of the efficiency and validity of the measurement.

In the comparison between the three different modes of administration for the respective three samples, it also emerged that, based on the results obtained in the test relative to the IRI scale and, specifically, in the Empathetic Activation sub-scale (useful to evaluate feelings of sympathy “oriented towards the other” and concern for unfortunate others) and in the Personal Discomfort subscale (useful to evaluate “self-oriented” feelings of personal anxiety and discomfort in tense interpersonal contexts), the administration through Alexa had a significant effect of greater empathetic activation and lower personal discomfort in the participants compared to the solitary compilation through the paper questionnaire, and a significant effect (even if of less weight compared to the previous one) with respect to the compilation through the operator.

With reference to the above-described effects produced by Alexa on the participants, the following factors are likely to be considered influential: (1) First of all, Alexa’s voice, evidently produced and synthesized with optimal prosodic characteristics by the manufacturer, aimed at encouraging a pleasant, fluid, and engaging interaction with the user. It is likely that the prosodic vocal component contributed to the production of an effect of involvement in the participant, an effect clearly more evident in the comparison with the self-filling mode, but equally significant compared to the administration provided by the in-person operator. (2) The second explanation can be traced back to the careful preliminary programming work of Alexa’s dialogic interaction, carried out during the skill construction phase. Since we wanted to avoid obtaining a result of simple and rigid vocal repetition of the succession of the test questions, particular attention was paid to the insertion of dialogic passages of colloquial tone and with the function preparatory to the presentation of the scales. Some expressions were identified and inserted with the precise purpose of showing attention to the interlocutor, for example where it was said that, in case of doubt or misunderstanding, he could at any time ask for the repetition of the sentence. Transitions from one block to another were thus made particularly smooth and, so to speak, “accompanied”. This design, aimed at obtaining a “natural involvement” of the participant in the task to be performed, likely produced the desired effect, which emerged in the form of a greater empathic activation in the face of the stress of the artificial device. (3) The third factor can be easily identified in the use of only the “voice means” as an instrument of administration, with the cancellation of possible interference due to the appearance of the interlocutor and non-verbal communication capable of influencing the user/candidate. All three factors that, in the conclusions of the study, we identified as decisive elements in favoring the empathic activation of the participants who answered the questions administered by the vocal assistant Alexa have found ample confirmation in numerous international studies and research efforts, although conducted on other specific topics. Namely, Weiste and Peräkylä asserted the value of the prosodic aspects of empathic communication in psychotherapy interaction: the evidence, in which therapists formulated their questions with attention to prosody (formulations characterized by prosodic continuity: constant rhythm and intonation, low and calm voice, and moderate intonation) was clearly successful, and patients felt more “understood” [85].

Also, Niculescu et al. [86] demonstrated the importance, in human–robotic interaction, of the verbal characteristics of spoken language, such as the tone of voice. Moreover, some linguistic suggestions, such as expressions of empathy and humor, positively influenced the quality of interaction with a social robot. At the end of their experiment, the robot programmed to be more extroverted, exuberant, and funny was the one that received the most approval from users in their evaluations of the overall interaction and its quality.

The humorous component (a scenario containing jokes) also improved the user’s perception regarding the evaluation of the empathy, personality (emotional impact), and behavior of the robot, as well as, of course, the ability to entertain. In relation to the results obtained with our experiment in which the vocal assistant Alexa was used, the contribution of Kraus seems to us significant also, who reported the hypothesis that “voice-only” communication increases empathic accuracy, compared to traditional communication involving the other senses [87]. “Vocal-only” communication allows the perceivers to focus their attention on the most active and precise channel of communication for transmitting emotions to others. The experiment illustrated showed that voice-alone communication elicits higher rates of empathic accuracy than vision alone and multisensory communication, through a greater focus on the linguistic and paralinguistic vocal cues (tone of voice, timbre, rhythm, time, intensity, emotional content, etc.) that accompany the speech.

The conclusions reported above are consistent with our experimental work, in which the hypothesis that “less is more” has been confirmed; namely, that speech-only communication, even if it involves only one of the modes of emotional expression, could significantly improve empathic accuracy with respect to multi-sense communication.

The correlations analysis of variables included in our study showed a clear association of Empathetic Activation with a positive and engaging perception of interaction with Alexa as a test administerer. Higher values of empathic activation were matched by equally high values in the subscales of Engagement, Closeness, Warmth, Perception of Human-likeness, and inversely correlated values of Perceived Eeriness and Personal Discomfort. The results obtained fit well within the recent construct of companion technologies as artefacts designed to evoke an emotional response, one similar to that from interacting with another human, and referring to the dimension of experience specific to companion technologies as empathetic [88]. Social cues such as small talk, self-disclosure, expert jargon, empathy, gossip, and politeness expressed in human-to-human conversations to build trust could also be used during conversations with artificial entities to gain the user’s trust [63,89,90,91]. Considering, finally, that the social desirability measure did not report significant associations with the assessment of interaction with the bot and the level of perceived engagement, nor with the subscales of Empathetic Activation and Personal Discomfort, it can be inferred that there was no response bias in the use of the self-report requests employed to test the study hypotheses.

## 5. Limitations and Future Studies

For this work, however, some limitations must be considered. First of all are those related to the characteristics of the sample, which was composed exclusively of young university students. Future studies should consider not only a numerical extension of the participants, but also a stratification of sampling that includes other age groups and different socio-demographic characteristics, such as different levels of education, digital skills, residence areas (e.g., urban areas, suburban, small towns), and professions. An additional limitation is in the instrument used for the assessment. An extension of the research should test the results through the use of different instruments that measure the same construct or direct the choice based on the assessment of different constructs and then use other measurement scales. Future studies may consider extending the assessment of interactive involvement in the use of voice assistance into different areas of health and wellness psychology interventions, as well as in sport and exercise. A further limitation of the work is having used only the female version of Alexa’s voice. Even though this version is the one most frequently used by users, and in any case is pre-set in devices with voice assistants, considering the recent option (July 2021) of switching voice gender (male/female) with the command “Alexa change your voice!”, we could have better compared the interactive effect and avoided possible gender bias in voice automation. In any case, a future extension of the study will have to take into account the gender variants of automated voice, including user preferences for voices, as well as any research on gender-neutral AI voices [92,93,94]. Given that Alexa’s language for communicating with participants was Italian, the expansion of the results of this study should also include a replication with other languages and participants of other nationalities. A final suggestion could be to carry out the comparison on all three groups in order to identify a possible distorting effect attributable to the active social desirability of the participants in the three administration conditions, as well as a comparative evaluation in the three conditions of concentration, ease, and perceived pressure using the ICEPP index.

## 6. Conclusions

This study presented two significant results: the administration of assessment tools by voice assistant can be considered substantially equivalent to the other modalities; and the interaction that took place during the administration showed an empathic activation effect significantly superior to the conditions of pencil–paper and operator-in-presence administration.

In terms of spin-offs, these results on the one hand encourage the design and testing of further professional assistance skills, while at the same time underlining that the objective scenario for the development of artificial communication technologies cannot only be the pursuit of a human-like (predominantly multimodal) naturalness of communication, but a deeper understanding of the special interactive effects that the manipulation of the properties of a single communication channel (such as voice) can produce would also be desirable.

## Figures and Tables

**Table 1:** Tested models and goodness-of-fit indices for the brief IRI.

	χ^2^	*df*	Δχ^2^	Δ*df*	CFI	TLI	RMSEA	ΔCFI	ΔTLI	ΔRMSEA
Models in each group										
Administration										
Pencil-paper	82.693	76			0.978	0.970	0.030			
Operator	89.074	76			0.969	0.958	0.041			
Alexa	119.479 *	76			0.953	0.954	0.066			
Global models										
Administration										
Configural	291.247 *	228	-	-	0.951	0.929	0.063	-	-	-
Metric	321.050 *	250	29.803	22	0.944	0.927	0.063	−0.007	−0.002	0.000
Scalar	355.377 *	272	34.327	22	0.943	0.920	0.065	−0.001	−0.007	0.002
Strict	432.393 *	318	77.016	46	0.944	0.918	0.060	0.001	−0.002	−0.005

Legend: *df* = degrees of freedom; χ**^2^** = chi square; Δχ**^2^** = difference in chi square; Δ*df* = difference in degrees of freedom; CFI = comparative fit index; TLI = Tucker–Lewis index; RMSEA = root–mean–square error of approximation; ΔCFI = difference in comparative fit index; ΔTLI = difference in Tucker–Lewis index; ΔRMSEA = difference in root–mean–square error of approximation. * = *p* < 0.001.

**Table 2:** Bivariate correlations.

	1	2	3	4	5	6	7	8	9	10	11	12	13	14	15
PT	1														
PD	−0.226 *	1													
EA	0.146	−0.213 *	1												
IM	0.070	0.021	−0.179	1											
IRI	0.396 **	0.325 **	0.412 **	0.365 **	1										
MAN	0.090	0.023	0.113	−0.092	0.077	1									
EXP	0.212 *	−0.024	0.263 **	−0.106	0.195	0.082	1								
EMO	0.091	-.098	0.104	0.025	0.068	0.084	0.217 *	1							
DES	0.260 **	−0.082	0.210 *	0.061	0.208 *	0.080	0.332 **	0.219 *	1						
ENG	0.231 *	−0.259 **	0.257 **	−0.042	0.105	0.094	0.168	0.324 **	0.056	1					
CLO	0.012	−0.310 **	0.268 **	−0.020	−0.031	0.103	0.173	0.301 **	0.130	0.309 **	1				
WAR	0.200 *	−0.313 **	0.336 **	−0.112	0.062	0.034	0.206 *	0.271 **	0.091	0.297 **	0.233 *	1			
COMP	0.179	−0.230 *	0.113	0.017	0.035	0.052	−0.069	−0.072	0.038	0.117	0.071	0.277 **	1		
HUM	0.242 *	−0.220 *	0.329 **	−0.074	0.156	0.077	0.332 **	0.264 **	0.160	0.284 **	0.321 **	0.023	−0.198 *	1	
EER	−0.036	0.277 **	−0.268 **	0.076	0.029	−0.007	−0.145	−0.170	−0.103	−0.263 **	0.000	−0.088	−0.252 *	−0.125	1

**. Correlation is significant at the 0.01 level (2-tailed). *. Correlation is significant at the 0.05 level (2-tailed). PT = Perspective Taking; PD = Personal Discomfort; EA = Empathetic Activation; IM = Imagination; IRI = Interpersonal Reactivity Index (general); MAN = Manipulativeness; EXP = Expressiveness; EMO = Emotionality; DES = Marlowe–Crowne Social Desirability; ENG = Engagement; CLO = Closeness; WAR = Warmth; COM = Competence; HUM = Human-likeness; EER = Eeriness.

## Data Availability

The data that support the findings of this study are available from the corresponding author upon reasonable request.
